# PPARs in Human Neuroepithelial Tumors: PPAR Ligands as Anticancer Therapies for the Most Common Human Neuroepithelial Tumors

**DOI:** 10.1155/2010/427401

**Published:** 2010-03-17

**Authors:** Elisabetta Benedetti, Renato Galzio, Barbara D'Angelo, Maria Paola Cerù, Annamaria Cimini

**Affiliations:** ^1^Department of Basic and Applied Biology, University of L'Aquila, 67100 L'Aquila, Italy; ^2^Department of Health Sciences (Neurosurgery), University of L'Aquila, 67100 L'Aquila, Italy

## Abstract

Neuroepithelial tumors represent a heterogeneous class of human tumors including benignant and malignant tumors. The incidence of central nervous system neoplasms ranges from 3.8 to 5.1 cases per 100,000 in the population. Among malignant neuroepithelial tumors, with regard to PPAR ligands, the most extensively studied were tumors of astrocytic origin and neuroblastoma. PPARs are expressed in developing and adult neuroepithelial cells, even if with different localization and relative abundance. The majority of malignant neuroepithelial tumors have poor prognosis and do not respond to conventional therapeutic protocols, therefore, new therapeutic approaches are needed. Natural and synthetic PPAR ligands may represent a starting point for the formulation of new therapeutic approaches to be used as coadjuvants to the standard therapeutic protocols. This review will focus on the major studies dealing with PPAR expression in gliomas and neuroblastoma and the therapeutic implications of using PPAR agonists for the treatment of these neoplasms.

## 1. Neuroepithelial Tumors

Human neuroepithelial tumors are classified according the World Health Organization (WHO). The incidence of central nervous system (CNS) neoplasms ranges from 3.8 to 5.1 cases per 100,000 in the population. Among neuroepithelial tumors, with regard to PPAR ligands, the most extensively studied are tumors of astrocytic origin and neuroblastoma. 

 Astrocytic tumors are classified as: (1) Astrocytoma (WHO grade II), (2) Anaplastic (malignant) astrocytoma (WHO grade III), (3) Glioblastoma multiforme (WHO grade IV); (4) Pilocytic astrocytoma noninvasive, (WHO grade I), (5) Subependymal giant cell astrocytoma (noninvasive, WHO grade I), (6) Pleomorphic xanthoastrocytoma (noninvasive, WHO grade I) [1–4]. 

 Malignant astrocytic tumors are the most common primary brain tumors. High-grade gliomas show high cellular proliferation rate and infiltrate the adjacent brain tissue [5]. They initially respond to radiation and, to a lesser degree, to chemotherapy; however, they invariably recur. The malignant gliomas with poor prognosis and fatal outcome are mainly represented by anaplastic astrocytoma and glioblastoma.

### 1.1. Anaplastic Astrocytoma (WHO Grade III)

Also known as malignant astrocytoma and high-grade astrocytoma, it may arise from a diffuse astrocytoma or may arise *de novo *without indication of a less malignant precursor [6]. Histologically, these tumors show increased cellularity, distinct nuclear atypia, and marked mitotic activity when compared with low-grade astrocytomas. Anaplastic astrocytomas possess an intrinsic tendency to progress to glioblastoma. The mean age at diagnosis is approximately 41 years. This tumor primarily affects the cerebral hemispheres. It has a high frequency of *TP53* mutations, which is similar to that of low-grade astrocytomas; chromosomal abnormalities are nonspecific. Many of the genetic alterations seen in anaplastic astrocytomas involve genes that regulate cell cycle progression [4]. The mean time to progression is 2 years. Positive predictive factors include young age, high performance status, and gross total tumor resection.

### 1.2. Glioblastoma (WHO Grade IV)

Also known as glioblastoma multiforme (GBM), it may develop from low-grade astrocytomas or anaplastic astrocytomas but more commonly it arises *de novo* without evidence of a less malignant precursor [7]. GBM, the most common malignant brain tumor (34%) in adults, is among the most lethal of all cancers [8]. Histologically, GBMs are anaplastic, cellular gliomas composed of poorly differentiated, often pleiomorphic astrocytic tumor cells with marked nuclear atypia and brisk mitotic activity. Typically, they affect adults and are preferentially located in cerebral hemispheres. Most patients with GBM survive less than 1 year, thus new therapeutic strategies are urgently needed [9, 10]. Genetic analyses suggest that there are two different types of glioblastoma: *de novo* glioblastoma, which arises from mutated neural stem cells or progenitor cells, and secondary glioblastoma, which arises from lower grade tumors. The secondary GBMs occur in younger patients [11–13]. The peak incidence occurs between the ages of 45 and 70 years. GBMs have been associated with more specific genetic abnormalities than any other astrocytic neoplasm, but none are specific. Amplification of the epidermal growth factor receptor locus is found in approximately 40% of primary GBMs but is rarely found in secondary glioblastomas; mutations of the *PTEN* gene are observed in 45% of primary GBMs and to a lesser extent in secondary glioblastomas [4]. Loss of heterozygosity (LOH) of chromosome 10 and loss of an entire copy of chromosome 10 are the most frequently observed chromosomal alterations.

### 1.3. Neuroblastoma

Neuroblastomas are paediatric tumors originating from neuroblasts in the developing peripheral nervous system. Most primary tumors (65%) occur within the abdomen, with at least half of these arising in the adrenal medulla. Other common sites of disease include the neck, chest, and pelvis. It is the most common extracranial solid tumor in childhood and the most frequently diagnosed neoplasm during infancy [14]. Neuroblastoma accounts for more than 7% of malignancies in patients younger than 15 years and around 15% of paediatric deaths [15]. The mortality is high due to rapid tumor progression to advanced stages. The genetic aberration most consistently associated with poor outcome in neuroblastoma is genomic amplification of *MYCN*, which occurs in roughly 20% of primary tumors and is strongly correlated with advanced stage of disease and treatment failure [14, 16, 17]. Deletions of the short arm of chromosome 1 (1p) can be identified in 25–35% of neuroblastomas. These deletions correlate not only with *MYCN *amplification, but also with advanced disease stage [18, 19]. However, the gene or genes within chromosome 1p involved in the pathogenesis of neuroblastoma have not been identified despite intensive investigation. It has been suggested that a strategy to halt the malignancy of these cells could be to induce them to differentiate towards mature neurons. Accordingly, several neuroblastoma differentiation protocols have been proposed, for instance treatment with phenyl acetate and retinoic acid [20]. The SH-SY5Y cell line was established from a high malignant tumor with no N-myc amplification [21]. Treatment of this cell line with phorbol esters leads to sympathetic neuronal differentiation with neurite outgrowth and increased synthesis of noradrenaline and expression of neuropeptide Y and growth-associated protein 43 (GAP-43) [22]. These effects are mediated by and dependent on PKC [20, 23, 24].

### 1.4. PPARs

The peroxisome proliferator-activated receptor (PPAR) family of nuclear receptors are ligand-activated transcription factors which have been implicated in different human pathologies. PPAR*γ* ligands are currently used for treatment of type II diabetes, PPAR*α* ligands are used to treat cardiovascular diseases [25–27]. After the isolation of PPAR*α* (NR1C1), in 1990 by Issemann and Green [28] as the nuclear receptor mediating peroxisome proliferation by peroxisome proliferators (PP) in rodent hepatocytes, two related isotypes, PPAR*β*/*δ* (NR1C2; referred to as PPAR*β*) and PPAR*γ* (NR1C3) have been characterized [29]. Since then, these receptors have been linked to many systemic and cellular functions ranging far beyond the process after which they were initially named. Like the other members of the superfamily, PPARs have a canonical nuclear receptor organization [30]. The DNA-binding domain, named the C domain, is highly conserved and its zinc finger domain is a common attribute of all members of the nuclear receptor (NR) superfamily. The C domain is linked to the C-terminal ligand-binding domain (LBD), named the E domain, by the hinge region, named the D domain. The LBD contains a ligand-dependent transactivation function referred to as AF-2 and comprises 12*α* helices and 4*β* sheets that fold to create a large hydrophobic cavity where ligands are buried [31]. In addition, the E domain offers the main surface for dimerization with the 9-cis retinoic acid receptor (RXR) as well as for interaction with regulatory proteins called cofactors. The N-terminal domain, named the A/B domain, is involved in ligand-independent regulation of receptor activity [32]; this domain harbors a weak-ligand-independent transactivation function, called AF-1. PPARs form heterodimers with the RXR and exhibit ligand-induced transcriptional regulatory activity through sequence-specific PPAR-responsive elements (PPRE) in their target genes [33]. Free PPARs may be associated with corepressors that inactivate the transcription function of the nuclear receptor. When the nuclear receptor is activated by a particular ligand binding to the ligand binding domain, this results in conformational changes to PPAR, and the receptor is released from binding with the corepressor. PPAR forms a heterodimeric complex with RXR and then recruits coactivator proteins. This complex binds to a PPRE on DNA and regulates transcription. Peroxisome proliferators, like fatty acids, modulate tissue-specific responses; for example, they stimulate the expression of enzymes involved in lipid catabolism, namely, the peroxisomal *β*-oxidation system [34, 35]. 

 PPARs exhibit a broad but isotype-specific tissue expression pattern which can account for the variety of cellular functions they regulate. PPAR*α* and *γ* transcripts appear late during fetal development of rat and mouse (day 13.5 of gestation), with a pattern of expression similar to their adult distribution, with the exception of the placenta tissue, where PPAR*γ* is abundantly expressed as early as E8.5 [36, 37]. It has been demonstrated that PPAR*γ* functions in the placenta are crucial for trophoblast terminal differentiation and consequently for placental vascularization and integrity [37]. In the adipose tissue, the two PPAR*γ* isoforms, *γ*1 and *γ*2, act in the brown and white tissues, respectively, to promote adipocyte differentiation and lipid storage, while the expression of the PPAR*γ*1 is preferentially shown in other tissues such as the gut or the immune cells [38]. PPAR*α* is expressed in tissues with high fatty acid catabolism such as the liver, heart, skeletal muscle, adrenal gland and pancreas, kidney, and intestine. In comparison with the two other isotypes, PPAR*β*/*δ* is expressed more ubiquitously and earlier during fetal development [39]. Its transcript is present in all organ tested, and it is often more abundant than the PPAR*α* and *γ* transcripts [40]. Shi et al. and Bastie have suggested the involvement of unliganded PPAR*β*/*δ* in modulating the expression and transcriptional activity of the other two PPARs [41, 42]. In addition, it has been shown that it is required for placenta development, in the control of cell proliferation and survival, especially in keratinocytes and enterocytes, and in the control of lipid metabolism, even though the underlying mechanisms still need investigations [43]. 

 For more than a decade, work on PPARs was driven by their important role in the regulation of cellular metabolism, PPAR*α* in tissues known for high *β*-oxidation rates such as liver, heart, muscle, and kidney [44], while PPAR*γ* was mainly studied for its adipogenic activity. At present, they are receiving growing attention for their involvement in the regulation of cell proliferation, death, and differentiation of both normal and malignant cells.

### 1.5. PPAR Ligands

PPARs are activated by a wide range of naturally occurring or metabolically produced lipids derived from the diet or from endogenous lipid molecules functioning in intracellular signalling pathways, which include saturated and unsaturated fatty acids and fatty acid derivatives such as prostaglandins and leukotrienes [45]. Whereas most natural agonists bind with a relatively weak affinity (in the order of *µ*molar concentration), some high-affinity endogenous ligands have been characterized [46, 47]. Interestingly, some ligands, including 15-deoxy-prostaglandin J2 (15d-PGJ2), associate irreversibly to the receptor through covalent binding [48]. The delivery of PPAR ligands to the nucleus, where the receptors reside, is achieved by different cellular fatty acid binding proteins (FABPs), which are thought to specifically interact with the three PPAR isotypes [49]. PPAR*α* agonists include both fibrates commonly used for the treatment of hypertriglyceridemia and the synthetic agonists WY 14,643 and GW7647. The best-characterized PPAR*γ* agonists are thiazolidinediones (TZDs), including pioglitazone and rosiglitazone, which have insulin sensitizing activity and are currently used for the treatment of type 2 diabetes (Actos and Avandia, respectively) [44]. There are a number of nonTZD based PPAR*γ* agonists, such as GW347845 and others that have been synthesized. 

 Apart from well-defined metabolic actions, PPAR*γ* agonists exhibit several antineoplastic effects [50] and induce apoptotic cell death in various malignant cell lineages, including liposarcoma [51], breast adenocarcinoma [52, 53], prostate carcinoma [54], colorectal carcinoma [55, 56], nonsmall-cell lung carcinoma [57], pancreatic carcinoma [58], bladder cancer [59], and gastric carcinoma [60]. 

 PPAR*β*/*δ* agonists include the prostacyclin PGI_2,_ oleic acid, and the agents, GW0742, GW501516 and GW7842.

## 2. PPARs in the Brain

All three PPAR isotypes are coexpressed in the rat CNS during late embryogenesis, with PPAR*β*/*δ* being the more abundantly and precociously expressed. The expression of the three PPAR isotypes peaks in the rat CNS between day 13.5 and 18.5 of gestation. Whereas PPAR*β*/*δ* remains highly expressed, the expression of PPAR*α* and PPAR*γ* decreases postnatally in the brain [61]. Both *in vitro* and *in vivo* observations show that PPAR*β*/*δ* is the prevalent isoform in the brain, found in all nervous cell types, whereas PPAR*α* is expressed at very low levels predominantly in astrocytes [30]. Acyl-CoA synthetase 2 (ACS2), an enzyme crucial for fatty acid activation and utilization, is regulated by PPAR*β*/*δ* at the transcriptional level, providing a simple measure of PPAR*β*/*δ* action [62]. ACS2 has a role in maturation of neurons (i.e., their cytodifferentiation and formation of neuronal connectivity); in addition, its over-expression in PC12 cells enhances internalization of fatty acids, namely oleic acid (OA), arachidonic acid (AA), and docosahexaenoic acid (DHA) and promotes neurite outgrowth [63]. These observations strongly suggest that PPAR*β*/*δ* participates in the regulation of lipid metabolism in the brain, a hypothesis further supported by the observation that PPAR*β*/*δ* null mice exhibit an altered myelination of the corpus callosum [64]. The fact that no cytoarchitectural alterations of cerebral cortex was described is in contrast with the results obtained by Michalik et al. and Cimini et al., demonstrating the existence of a close correlation between PPAR*β*/*δ* and ACS2 in PPAR*β*/*δ* null mice brain and in the rat cortical neurons, respectively [65, 66]. However, the study from Peters et al. is a mainly *in situ* study and may be only in apparent contrast with other authors because it does not exclude that neuronal function and competence may be impaired in PPAR*β*/*δ* null mice [64]. 

 All PPARs have been described in the adult and developing brain and spinal cord [67–69]. While PPAR*β*/*δ* has been found in neurons of numerous brain areas, PPAR*α* and PPAR*γ* have been localized to more restricted brain areas [67, 68]. The localization of PPARs has been also investigated in purified cultures of neural cells. Previous studies have reported that PPAR*β*/*δ* is strongly expressed in immature oligodendrocytes (OL) where its activation promotes differentiation; PPAR*γ* is mainly present in microglia, while astrocytes possess all three PPAR isotypes, although to different degrees depending on the brain area and animal age [70–74]. The role of PPARs in the CNS has mainly been related to lipid metabolism but these receptors have been recently implicated in neural cell differentiation and death as well as in inflammation and neurodegeneration [36]. PPAR*α* has been suggested to be involved in astrocyte maturation and differentiation both in primary adult mouse astroglial cells and in adult neural stem cells (NSC) [75–77]. In addition, this isotype has been suggested to be involved in acetylcholine metabolism and oxidative stress defense [68, 78]. 

 PPAR*γ*, besides playing a role in early phases of oligodendrocyte differentiation, has been mainly studied in relation to inflammation, cancer, and neurodegeneration [79]. Concerning PPAR*β*/*δ*, its involvement in neuronal differentiation and in CNS development has been suggested by Basu-Modak et al., Michalik et al., Braissant and Whali, on the basis of its high levels in rat neural tube, in reaggregated neural cell cultures and in adult CNS [62, 65, 80]. Roles for PPAR*β*/*δ* in the regulation of pain sensation and transmission in adult spinal cord [69] and in learning and memory in mouse hippocampus and in entorhinal cortex have been proposed [67, 69]. In a previous work we showed that PPAR*β*/*δ* is the main isotype present in primary cultures of rat cortical neurons, where it seems necessary for neuronal maturation together with the reduction of PPAR*γ* expression and the activation of PPAR*α*; PPAR*β*/*δ*, in fact, is gradually increased and activated during neuronal maturation, and this increase correlates with the expression of its target gene ACS2 [81].

## 3. PPARs in Neuroepithelial Tumors

Among neuroepithelial tumors, gliomas, especially glioblastoma and neuroblastoma, have been the most extensively studied as to regard PPARs, and in this context, the PPAR*γ* isotype was the most extensively studied.

### 3.1. PPARs and GBM

Glioblastoma expresses all three PPAR isotypes both *in vitro* and *in situ*. The majority of the studies performed on this neoplasm, both on rat and human gliomas, reported on the antiproliferative activity of different PPAR*γ* ligands, both natural and synthetic, by promoting apoptotic cell death or by increasing reactive oxygen species production [82–86]. PPAR*γ* has been identified in transformed neural cells of human origin, and PPAR*γ* agonists have been shown to decrease cell proliferation, stimulate apoptosis, and induce morphological changes as well as expression of markers typical of a more differentiated phenotype in glioblastoma and astrocytoma cell lines [87–89]. These findings have more recently been confirmed in glioblastoma primary cultures; treating primary cultures of gliobalstoma cells with natural or synthetic PPAR*γ*  ligands  decreases the expression of markers of undifferentiated stages, such as CD133, nestin and fibronectin, while increasing the expression of differentiation markers such as A2B5, GFAP, *β*-catenin, and N-cadherin. Conjugated linoleic acid (CLA) and the PPAR*γ* synthetic agonist GW347845 also suppress proliferation and induce apoptosis in primary cultures of glioblastoma cells [90]. Consistent with growth inhibition, both ligands downregulate cyclinD1 and CDk4 protein levels, while inducing the transcription of the tumor suppressor gene PTEN. Both CLA and PPAR*γ* agonist lead to a significant decrease of the VEGF isoforms and NOSII, thus indicating that even in glioblastoma  PPAR*γ* is able to inhibit the angiogenic pathways [90]. It has also been reported that ciglitazone induces apoptosis in four human glioblastoma cell lines by decreasing cyclin D1 and Bcl-2 proteins and increasing p27 and p21 proteins [91, 92]. Finally, there is some evidence to suggest that TZDs are potent inhibitors of glioma cell migration and brain invasion largely by transcriptional repression of TGF-*β* [93]. This is particularly important because TGF-*β* is an immunosuppressive cytokine that has been shown to play a major role in the malignant phenotype of gliomas [94]. Furthermore, inhibition of TGF-*β* signaling restores immune surveillance and is associated with improved survival in a glioma model [95]. 

 Apoptosis-based therapies gained interest as promising experimental treatment strategies since direct induction of apoptotic cell death can overcome many of the classical resistance mechanisms such as activated DNA repair or detoxification. The death ligand TRAIL/Apo2L might be a useful tool to trigger apoptosis in cancer, since TRAIL kills tumor cells of diverse cellular origin without severe toxic side effects [96, 97]. However, despite the common expression of death receptors, not all glioblastoma cells are susceptible to TRAIL, due to intracellular blockage of apoptotic signalling cascades. A group of PPAR*γ*-modulating agents sensitize tumor cells to TRAIL-induced apoptosis [98]. It has been reported that glioblastoma cells are sensitized to TRAIL-induced apoptosis by troglitazone via various mechanisms. Troglitazone lead to a marked down-regulation of the antiapoptotic proteins FLIP and Survivin. Moreover, in some cell lines, the cell surface expression of agonistic and antagonistic TRAIL receptors was altered towards a higher susceptibility to death receptor-induced apoptosis. Troglitazone might counteract the capability of tumor cells to become resistant to apoptosis by modulating the apoptotic machinery at different levels [98]. 

 It should be noted that only PPAR*γ* ligands have been described as antiproliferative compounds in gliomas; no effects have been reported for other PPAR ligands. However, some evidences, obtained by us [99] in human gliomas at different grades of malignancy, strongly indicate an upregulation of PPAR*α* and its direct relationship with malignancy grade, suggesting that PPAR*α* antagonists can be used to halt malignancy, and suggesting that in some cases dual PPAR agonists should be carefully used. 

 Taken together, these data reported in glioma cells indicate that PPAR*γ* activation by both synthetic and natural ligands, results in cell cycle arrest, apoptosis promotion, inhibition of cell migration and invasion as well as suppression of antiapoptotic proteins, induction of differentiation markers, thus suggesting their potential use in the formulation of new therapeutic strategies against this neoplasm and recurrences.

### 3.2. PPARs and Neuroblastoma

Neuroblastoma cells express all three isotypes of PPARs. PPAR*γ* is present in neuroblastoma cell lines [100], as well as in primary neuroblastoma cell culture [50]. Few studies report the expression of PPAR*α* at mRNA or protein level in human neuroblastoma cell lines [101] and data on the expression of PPAR*β*/*δ* in neuroblastomas are scarce [102]. To assess the roles of PPARs on neuroblastoma, most studies evaluate the impact of their natural or synthetic ligands on cell proliferation, death and differentiation. The putative natural PPAR*γ* agonist, 15d-PGJ2, inhibits cellular growth, decreases cellular viability and induces apoptosis in human neuroblastoma cells *in vitro* [100, 103, 104], although some effects have been demonstrated to be PPAR*γ*-independent [105]. Rodway et al. [103] showed that the PPAR*α* agonist WY-14643 has no effect on the growth of the IMR32 neuroblastoma cell line, whereas PGJ2 induces growth inhibition in the same neuroblastoma cells. This occurs through programmed cell death type II or autophagy, and the serum lysolipid, the lysophosphatidic acid (LPA), is responsible for modulating this cellular response. In the neuroblastoma cell line ND-7, the same group showed that the degree of PPAR*γ* activation induced by PGJ2 is modulated through an interaction with the retinoblastoma protein (Rb) and histone deacetylase [106]. A combination therapy consisting of PGJ2 and the histone deacetylase inhibitor trichostatin A enhances the growth inhibition effects and is therefore proposed as a promising new strategy in the treatment of neuroblastoma. It should be noted that the effects of 15d-PGJ2 can also depend on its action on the NF*κ*B pathway [107]. Valentiner et al. [108] tested four synthetic PPAR*γ* TZD agonists (ciglitazone, pioglitazone, troglitazone, rosiglitazone) and reported their *in vitro* effects on cell growth of seven human neuroblastoma cell lines (Kelly, LAN-1, LAN-5, LS, IMR-32, SK-N-SH, and SH-SY5Y). All TZDs inhibited cell growth and viability of the cells in a dose-dependent manner, whereas the effectiveness of the single drugs was strongly different among cell lines. Similar results for ciglitazone and rosiglitazone have been reported [100, 109]. Cellai and colleagues [109] showed that high concentrations of rosiglitazone *in vitro* significantly inhibit cell adhesion, invasiveness, and apoptosis in SK-N-AS, but not in SH-SY5Y human neuroblastoma cells. The authors argued that this effect may be related to cellular differences in PPAR*γ* transactivation. We have recently demonstrated [110] that PPAR*β* */* *δ* agonists, both natural and synthetic (oleic acid and GW0742, respectively), are able to induce cell cycle arrest in G1 phase and neuronal differentiation in human neuroblastoma cell line SH-NH-5YSY by increasing p16 levels and decreasing cyclin D1 levels as well as by inducing the expression of neuronal differentiation markers and downregulating Tr*κ*B full length expression. In the case of neuroblastoma, both PPAR*γ* and PPAR*β* */* *δ* ligands showed antiproliferative effects but it should be noted that PPAR*γ* activation results in apoptosis promotion, while PPAR*β* */* *δ* activation results in cell cycle arrest and neuronal differentiation, thus suggesting the possibility to use dual agonists to counteract tumor progression and recurrences.

## 4. Future Perspectives

The majority of malignant neuroepithelial tumors have poor prognosis and do not respond to conventional therapeutic protocols. Studying and validating both natural and synthetic PPAR ligands may represent a starting point for the formulation of new therapeutic approaches to be used as coadjuvants to the standard therapeutic protocols. Another point to be considered is the targeting efficiency of these new drugs. The progress in the understanding of the biology and genetic of neuroepithelial tumors together with the use of truly manipulable experimental models, now offer real opportunities for the development of effective targeted therapy. Despite significant gaps in our understanding, a wealth of information now exists about the clinical and biological behaviours of these tumors, the genetic pathways involved in tumorigenesis, and the nature and role of signature alterations in these pathways. The challenge now is to integrate this knowledge in an interdisciplinary way to fully understand these diseases, particularly how their signature heterogeneity contributes to their intractability in order to design efficient drugs delivered exclusively to malignant cells.
