# Corrigendum to “PPARs in Human Neuroepithelial Tumors: PPAR Ligands as Anticancer Therapies for the Most Common Human Neuroepithelial Tumors”

**DOI:** 10.1155/2019/4309068

**Published:** 2019-07-21

**Authors:** Elisabetta Benedetti, Renato Galzio, Barbara D'Angelo, Maria Paola Cerù, Annamaria Cimini

**Affiliations:** ^1^Department of Basic and Applied Biology, University of L'Aquila, 67100 L'Aquila, Italy; ^2^Department of Health Sciences (Neurosurgery), University of L'Aquila, 67100 L'Aquila, Italy

The article titled “PPARs in Human Neuroepithelial Tumors: PPAR Ligands as Anticancer Therapies for the Most Common Human Neuroepithelial Tumors” [1] was found to contain material in Sections 1, 2, and 3 from published work and to have missing attributions and errors in citations. The articles are as follows:

- M. T. Heneka and G. E. Landreth, “PPARs in the brain,” Biochimica et Biophysica Acta, vol. 1771, no. 8, pp. 1031–1045, 2007. https://doi.org/10.1016%2fj.bbalip.2007.04.016. [2] (Cited as reference [36]).
- Lars Tatenhorst, Eric Hahnen, and Michael T. Heneka, “Peroxisome Proliferator-Activated Receptors (PPARs) as Potential Inducers of Antineoplastic Effects in CNS Tumors,” PPAR Research, vol. 2008, Article ID 204514, 9 pages, 2008. https://doi.org/10.1155/2008/204514. [3] (not cited).
- Markus P. Kummer and Michael T. Heneka, “PPARs in Alzheimer's Disease,” PPAR Research, vol. 2008, Article ID 403896, 8 pages, 2008. https://doi.org/10.1155/2008/403896. [4] (not cited).
- J. N. Feige, L. Gelman, L. Michalik, B. Desvergne, and W. Wahli, “From molecular action to physiological outputs: peroxisome proliferator-activated receptors are nuclear receptors at the crossroads of key cellular functions,” Progress in Lipid Research, vol. 45, no. 2, pp. 120–159, 2006. https://doi.org/10.1016%2fj.plipres.2005.12.002. [5] (cited as reference [30]).
- A. Cimini, E. Benedetti, L. Cristiano et al., “Expression of peroxisome proliferator-activated receptors (PPARs) and retinoic acid receptors (RXRs) in rat cortical neurons,” Neuroscience, vol. 130, no. 2, pp. 325–337, 2005. https://doi.org/10.1016%2fj.neuroscience.2004.09.043. [6] (cited as reference [81]).
